# Towards planning of osteotomy around the knee with quantitative inclusion of the adduction moment: a biomechanical approach

**DOI:** 10.1186/s40634-021-00324-3

**Published:** 2021-06-11

**Authors:** Margit Biehl, Philipp Damm, Adam Trepczynski, Stefan Preiss, Gian Max Salzmann

**Affiliations:** 1 Fraunhofer Institute for Biomedical Engineering IBMT, Joseph-von-Fraunhofer-Weg 1, 66280 Sulzbach, Germany; 2 Charité – Universitätsmedizin Berlin, corporate member of Freie Universität Berlin, Humboldt-Universität zu Berlin and Berlin Institute of Health, Berlin, Germany; 3 Lower Extremity Orthopaedics, Musculoskeletal Center, Schulthess Clinic, Zurich, Switzerland; 4 Gelenkzentrum Rhein-Main, Wiesbaden, Germany

**Keywords:** High tibial osteotomy, Supracondylar osteotomy, Medial compartment force ratio, Leg alignment, Biomechanics of osteotomy, Adduction moment

## Abstract

**Purpose:**

Despite practised for decades, the planning of osteotomy around the knee, commonly using the Mikulicz-Line, is only empirically based, clinical outcome inconsistent and the target angle still controversial. A better target than the angle of frontal-plane static leg alignment might be the external frontal-plane lever arm (EFL) of the knee adduction moment. Hypothetically assessable from frontal-plane-radiograph skeleton dimensions, it might depend on the leg-alignment angle, the hip-centre-to-hip-centre distance, the femur- and tibia-length.

**Methods:**

The target EFL to achieve a medial compartment force ratio of 50% during level-walking was identified by relating in-vivo-measurement data of knee-internal loads from nine subjects with instrumented prostheses to the same subjects’ EFLs computed from frontal-plane skeleton dimensions. Adduction moments derived from these calculated EFLs were compared to the subjects’ adduction moments measured during gait analysis.

**Results:**

Highly significant relationships (0.88 ≤ *R*^2^ ≤ 0.90) were found for both the peak adduction moment measured during gait analysis and the medial compartment force ratio measured in vivo to EFL calculated from frontal-plane skeleton dimensions. Both correlations exceed the respective correlations with the leg alignment angle, EFL even predicts the adduction moment’s first peak. The guideline EFL for planning osteotomy was identified to 0.349 times the epicondyle distance, hence deducing formulas for individualized target angles and Mikulicz-Line positions based on full-leg radiograph skeleton dimensions. Applied to realistic skeleton geometries, widespread results explain the inconsistency regarding correction recommendations, whereas results for average geometries exactly meet the most-consented “Fujisawa-Point”.

**Conclusion:**

Osteotomy outcome might be improved by planning re-alignment based on the provided formulas exploiting full-leg-radiograph skeleton dimensions.

**Supplementary Information:**

The online version contains supplementary material available at 10.1186/s40634-021-00324-3.

## Introduction

Osteotomy around the knee is a proven surgical intervention to counterbalance overloaded knee compartments. However, despite decades of experience, the optimal correction angle is still controversial and outcome inconsistent. Whereas, in case of valgus precondition, correction recommendations are ranging around neutral alignment [52], the vast majority of long-term-outcome studies revealed that for varus precondition the postoperative “**M**echanical axis **A**ngle (**MA**)” of leg alignment should be at least 3° valgus for long-term-success. Apart from that, the relationship between postoperative MA and individual outcome was found surprisingly inconsistent [45, 53, 60].

By contrast, epidemiological [22], biomechanical [30] and some few outcome studies [14, 33] indicated that even slight valgus alignment might be harmful, and load experiments using cadaveric knees revealed that more pressure is applied to the lateral knee compartment even at neutral alignment of the MA, suggesting that overcorrection into valgus alignment might be unnecessary [1, 48]. Recent approaches thus aimed at MAs of less than 3° valgus – long-term-results pending [15, 36].

Altogether, a lack of scientific understanding, especially regarding the dynamic gait situation, impedes the optimization of osteotomy planning [3, 45], even though the importance of this dynamic situation is definitely recognized: As known for decades, the outcome of osteotomy around the knee substantially worsens with increasing “external **K**nee **A**dduction **M**oment (**KAM**)” measured during gait-analysis, despite comparable MA [51]. The KAM is the external frontal-plane torque affecting the knee joint, primarily evoked by the ground-reaction-force acting towards the weight-bearing leg.

Recent in vivo load measurements confirmed that the KAM influences the force distribution between both knee compartments [21, 42, 66]: In nine subjects with instrumented knee prostheses, the KAM was found highly correlated with the knee-internal adduction moment, which balanced about two-thirds of the KAM [66], and outstanding linear relationship was found between the KAM and the percentage of axial knee force transferred to the medial compartment over the complete stance phase of walking gait [42].

Oddly enough, the KAM correlates only moderately and very inconsistently with the MA, the actual target measure for planning osteotomy [37, 51, 62], and no significant relationship was found between the average change of the KAM and the average change of the MA by osteotomy for various patient populations [40]. Above findings entailed attempts and proposals to account for the KAM when planning osteotomy [37, 62]. To our knowledge, however, nobody so far has proposed a method for including the KAM into osteotomy planning quantitatively, thus individualizing target angles of leg alignment. It is the main topic of this work.

## Methods

### Theoretical basics

The KAM can essentially be calculated by multiplying the frontal-plane component of the ground-reaction-force with the perpendicular distance of its load-bearing-axis to the knee centre. This distance is denoted as “**E**xternal **F**rontal plane **L**ever arm (**EFL**)” below. EFL is largely constant over the stance phase of gait and the key driver for the KAM magnitude, thus has been proposed as a more suitable measure for planning osteotomy than the MA [37].

**Fig. 1:**
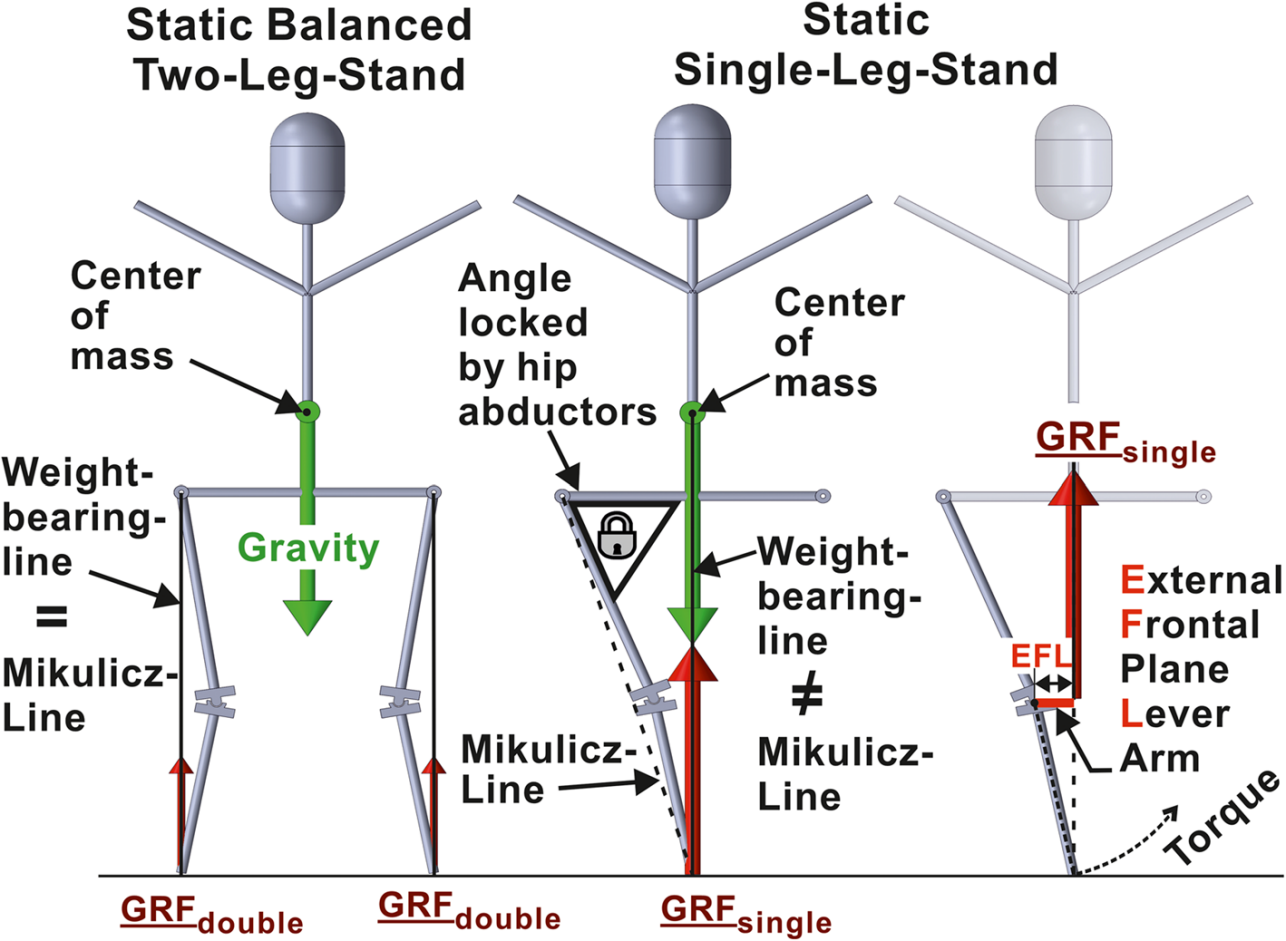
External forces and torques acting on the knee joint during static standing situations. During static balanced two-leg stand, the body is contacting the ground at two positions, where a “Ground-Reaction-Force (GRF)” of about half gravity is acting in the direction of the leg’s Mikulicz-Line, the action line of the GRF being usually conceived as the weight-bearing-line. At static single-leg-stand, only one contiguous area is contacting the ground perpendicular below the center of mass. The GRF now has the same action line and magnitude as gravity, consequently does not correspond to the Mikulicz-Line. Hence, in single-support situations not only a different weight-bearing-line applies, but also the GRF acting on the distal end of the tibia for gravity compensation is substantially higher than in static balanced two-leg-situations. The GRF, pulling the distal end of the tibia upwards, induces a knee torque (essentially the KAM), which influences the force distribution between both knee compartments. This torque is physically the same as if the GRF pulled at the end of a lever arm fixed to the proximal tibia, which extends from the knee center perpendicular towards the GRF axis (right). Its length, the External Frontal plane Lever arm (EFL), clearly depends on leg alignment, but is a physically more accurate predictor of the KAM than the MA, as it depends on the distance between both hip centers, the femur- and tibia-length, too

Evidently, size and direction of the EFL depend on the position of the leg’s frontal-plane load-bearing-axis relative to the knee centre. Orthopaedic treatises commonly equalize this load-bearing-axis to the “Mikulicz-Line”, which connects the talotibial joint centre with the hip centre [59]. This is justified approximation for the static balanced two-leg-stand (Fig. 1 left), but not for everyday dynamic situations like level-walking, where eighty-two percent of time are spent in dynamic single-leg-support [17], with much higher in-vivo knee loads [41] and the load-bearing-axis positioned much more medial [3, 62]. Hence, the average load-bearing-axis of level-walking, and not the Mikulicz-Line, should be shifted to one defined position when planning osteotomy around the knee.

Exploiting various publications on gait biomechanics [19, 44, 46, 49, 50, 64], we found that the temporally averaged frontal-plane load-bearing-axis of level-walking is virtually meeting the mid-position between both hip centres for comfortable walking speed (Appendix A.1). It therefore resembles the weight-bearing-line of the static single-leg-stand, which will be stable, if the body centre of mass is perpendicular above the base of support [35], with the ground-reaction-force counteracting gravity vertically near midline pelvis (Fig. 1 mid). Hence, the average EFL of level-walking approximately corresponds to the distance between the sagittal plane and the knee centre, i.e. the EFL, at static single-leg-stand. This single-leg-stand-EFL, again, depends not only on the MA, but also on the hip-centre-to-hip-centre distance, the femur- and tibia-length (Fig. 1 right), thus being a physically more accurate predictor for KAM and compartmental force distribution than the MA.

**Fig. 2:**
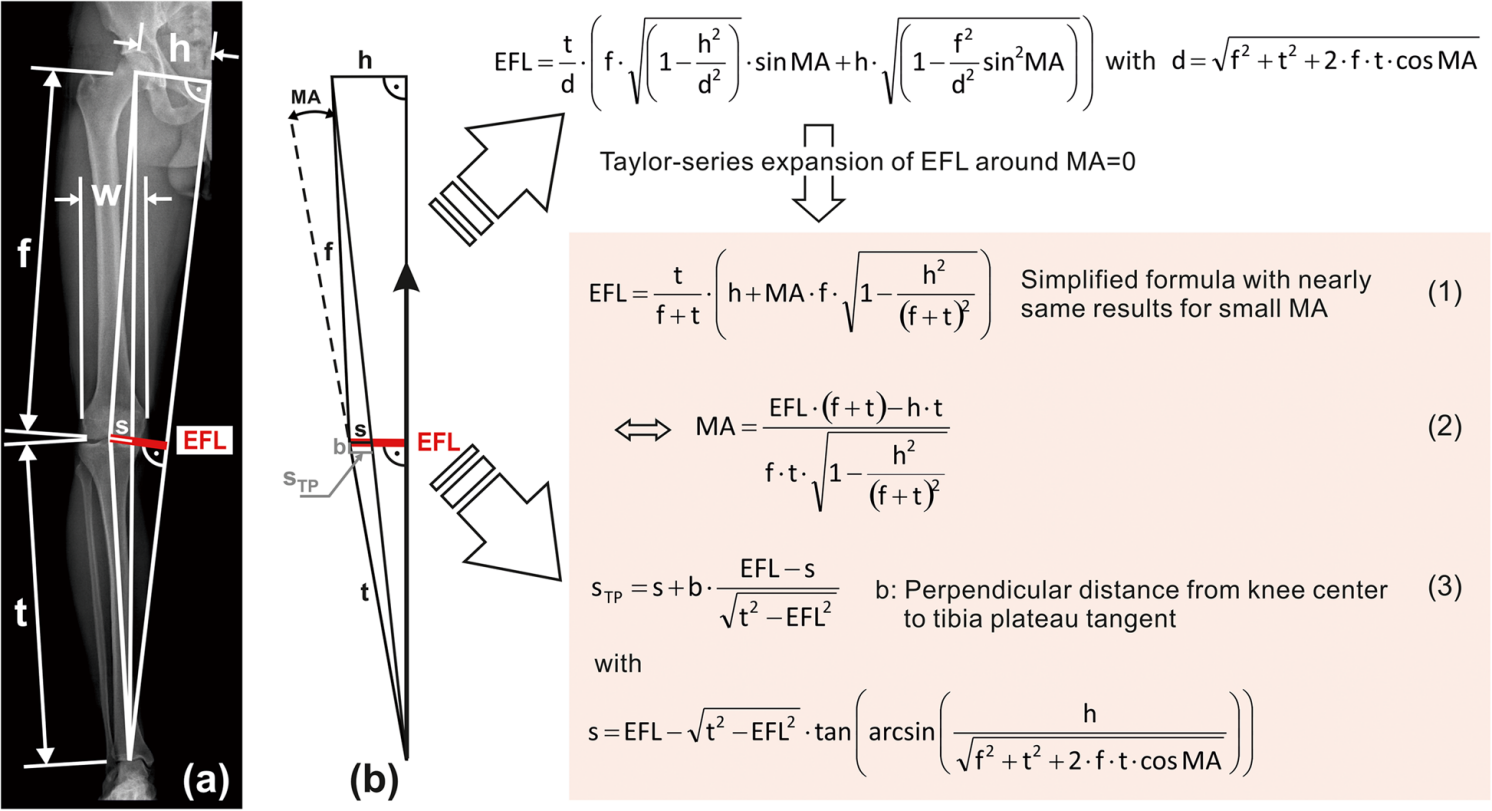
Calculation of the adduction moment’s External Frontal-plane Lever arm (EFL) from skeleton dimensions. The relevant straight-line segments (**a**) are depicted once again in counterclockwise rotated posture (**b**), which represents the static single-leg-stand situation, with the ground-reaction-force (arrow) acting bottom-up vertically along midline pelvis, and with the tibia-plateau ideally parallel to the ground after osteotomy. The highlighted formulas derived from (**b**) allow conversion of the MA (in radians) to the EFL and vice versa including further skeleton dimensions, as well as calculation of the lateral distance s_TP_ from the Mikulicz-Line to the knee centre on the tibia plateau. The large-arrow-marked formulas are directly derived from the trigonometric relationships between lengths and angles in (**b**). Equation (1) results from a usual mathematical approximation of the first, complex formula by a simpler polynomial

The EFL can be modified by osteotomy around the knee, thus surgically changing the MA. However, due to the higher physical accuracy, we propose that the target parameter for osteotomy should not be the MA, but the EFL of the KAM at static single-leg-stand. This single-leg-stand-EFL, again, can be calculated from static skeleton dimensions extractable from any standard full-leg radiograph with pictured symphysis pubis (Eq. (1) Fig. 2), wherefrom the following dimensions can be measured:

- Distance h from hip centre to midline pelvis, approximately given by the ipsilateral cranial edge of the symphysis pubis. If the patient’s frontal plane cannot be aligned exactly parallel to the image plane, half the distance between both hip centres should be measured from a radiograph of the pelvis.
- Femur length f from hip centre to knee centre.
- Tibia length t from talotibial joint centre to knee centre.
- Distance w between medial and lateral femur epicondyle.
- Distance b from knee centre to tibia plateau tangent.

The EFL should be the target parameter for osteotomy, wherefrom the individual target MA (Eq. (2) Fig. 2) and the lateral distance of the Mikulicz-Line to the knee centre on the tibia plateau s_TP_ (Eq. (3) Fig. 2) can be calculated.

For balanced compartmental morbidity risk, we presumed the optimal EFL achieved, if the same average contact pressure (force per contact area, stress) is applied to the medial and lateral compartment, as heightened average and maximum contact stress is associated with increased joint morbidity [55, 56]. Exploiting published pressure- and contact-area-measurements in twenty-three cadaveric knees [1, 48, 52, 65], we found equal pressure in both compartments when the axial force is distributed fifty-fifty (Appendix A.2). Hence, our approach aims at a “**M**edial compartment **F**orce **R**atio (**MFR**)”, which is the percentage of axial knee-internal force transferred to the medial compartment in vivo, of 50% over the complete stance phase of gait.

The MFR for any defined period with varying knee loads can be derived to (Appendix A.3)

4 $${\text{MFR}} = \left( {\frac{1}{2} + {\text{c}} \cdot \frac{{\text{IFL*}}}{{\text{w}}}} \right) \cdot 100$$

with c being the ratio of the femur-epicondyle-distance w to the femur-condyle-distance, and IFL* given by

5 $${\text{IFL*}} = \frac{{\sum\limits_{{\text{i}}} {{\text{M}}_{{{\text{Yi}}}} } }}{{\sum\limits_{{\text{i}}} {{\text{F}}_{{{\text{Zi}}}} } }}$$

Therein, -F_Zi_ is the knee-internal force component along the tibia axis in cranio-caudal direction at an instant i of the defined period, and -M_Yi_ is the simultaneous knee-internal torque in the frontal-plane, which shifts surplus load to the medial compartment and unloads the lateral one by the same amount. Summation (**∑**) is performed over all (isochronously distributed, discrete) instants i of the period.

IFL* can be conceived as the average weighted distance between the action line of the axial knee-internal force (caused by gravity and muscle forces) and the knee centre in the frontal-plane for the defined period (Appendix A.3), thus representing a “knee-**I**nternal **F**rontal plane **L**ever arm (**IFL**)”. According to Eq. (4), the fifty-percent-MFR for the defined period will be achieved, if IFL*/w equals zero. If the knee-width-normalized IFL*/w can be linearly related to the likewise knee-width-normalized EFL/w calculated from frontal-plane skeleton dimensions, the one EFL/w for balanced compartmental forces over this period can be identified from the intercept.

### Data sources

To find this relationship, skeleton data and in-vivo-measurement data of forces and torques within the knee during static balanced two-leg-stand, static single-leg-stand and level-walking (Appendix A.4), originating from nine subjects with instrumented knee prostheses [8, 9, 11–13], were exploited. Data had been collected before by a further study approved by the ethics committee of the Charité-Universitätsmedizin Berlin “(EA4/069/06) and registered at the ‘German Clinical Trials Register’ (DRKS00000606)” [13]. Within this study, all subjects provided written informed consent concerning use of their data.

### Proceeding

As detailed in Appendix A.5, for each subject various IFL* were computed from the time-variable forces F_Zi_ and torques M_Yi_ measured in vivo by the subject’s instrumented knee prosthesis (Eq. (5)). IFL* calculated for the complete stance phase of level-walking with the ipsilateral foot contacting the ground, the relevant period for osteotomy, is denoted as IFL_LW_ below. IFL* was further calculated for three sub-periods of the stance phase characterized by increasing compartmental force differences, as well as for the static single-leg-stand and the static balanced two-leg-stand, both static situations analysed over 1.9 s. From all these various IFL* values, associated MFR values were calculated using Eq. (4) (with subject-specific w and arbitrary, but consistent c).

Moreover, each subject’s individual EFL was calculated from its frontal-plane skeleton dimensions using Eq. (1) (Fig. 2) (Appendix A.5). As the ground-reaction-force at static single-leg-stand is approximately one times “**B**ody **W**eight (**BW**)”, the individual “**K**nee **A**dduction **M**oment at **s**tatic single-leg-stand (**KAM**_**S**_)”, in the usual unit [%BWHt], resulted from the subject-specific EFL and the subject’s “body **H**eigh**t** (**Ht**)” [42] as follows:

6 $$\begin{aligned}{{\text{KAM}}_{{\text{S}}} } & { = {{{\text{EFL}} \cdot {1} \cdot {\text{BW}}} \mathord{\left/ {\vphantom {{{\text{EFL}} \cdot {1} \cdot {\text{BW}}} {\left( {{\text{BW}} \cdot {\text{Ht}}} \right) \cdot {1}00}}} \right. \kern-\nulldelimiterspace} {\left( {{\text{BW}} \cdot {\text{Ht}}} \right) \cdot {1}00}}} \\ {} & { = {{{\text{EFL}}} \mathord{\left/ {\vphantom {{{\text{EFL}}} {{\text{Ht}} \cdot {1}00\,\;\;\;\;\;\;\;\;\;\;\;\;\;\;\;\;\quad }}} \right. \kern-\nulldelimiterspace} {{\text{Ht}} \cdot {1}00\,\;\;\;\;\;\;\;\;\;\;\;\;\;\;\;\;\quad }}} \end{aligned}$$

The KAM_S_ values therefore are equal to EFL in percentage of body height.

For knee width normalization, each subject's EFL and IFL*-values were divided by the subject-specific femur epicondyle distance w.

To compare the compartmental load distributions in vivo, the subjects' MFRs of the stance phase of level-walking were related to the MFRs of the static balanced two-leg- and single-leg-stand.

To test whether EFL is a significant predictor for KAM and MFR, the subjects' KAM_S_ (= EFL in %Ht) values were related to the same subjects’ published KAM peaks measured during gait analysis [42], and the subjects’ various MFR values were related to their EFL/w values. For comparison, the same correlations were tested with MA instead of EFL.

To find the target EFL/w for osteotomy, the subjects’ EFL/w values were related to their IFL_LW_/w values, and, for comparison, to their IFL*/w values from static single-leg-stand, denoted as IFL_SLS_/w below. The intercepts of the two highly significant linear correlations of EFL/w with IFL_SLS_/w and IFL_LW_/w delivered the target proportions, which are related to an MFR of 50% during static single-leg-stand and the stance phase of level-walking, respectively. From the universal target proportion EFL/w for the complete stance phase of level-walking, the individual target EFL for an osteotomy patient can be calculated by multiplying with the patient’s femur epicondyle distance w. This EFL inserted in Eq. (2) (Fig. 2) delivers the individual target MA for osteotomy in radians. Inserting EFL and MA in Eq. (3) (Fig. 2) finally yields the individual medio-lateral target distance of the Mikulicz-Line from the knee centre on the tibia plateau s_TP_. With known diameter of the tibia plateau and known lateral distance from the knee centre to the tibia plateau centre, s_TP_ can be easily expressed in percent of the tibia plateau diameter measured from the medial edge, too.

For evaluation regarding anthropometrically established body proportions, the resulting correction formulas were applied to fictive patients with realistic skeleton geometries, presetting various figures by varying hip-centre-to-hip-centre distance and body height. For each body height, average gender-dependent f, w, t, and b was calculated by using published average dimensions and proportions (Appendix A.6). Average figures were defined by combining average German body height [63] with average hip-centre-to-hip-centre distance measured from British patients’ radiographs (75 male, 75 female) [2]. Combining average body height with the measured hip-centre-to-hip-centre extremes delivered the margin figures “lanky man” and “stocky woman” (Table 3).

### Data analysis

**Fig. 3:**
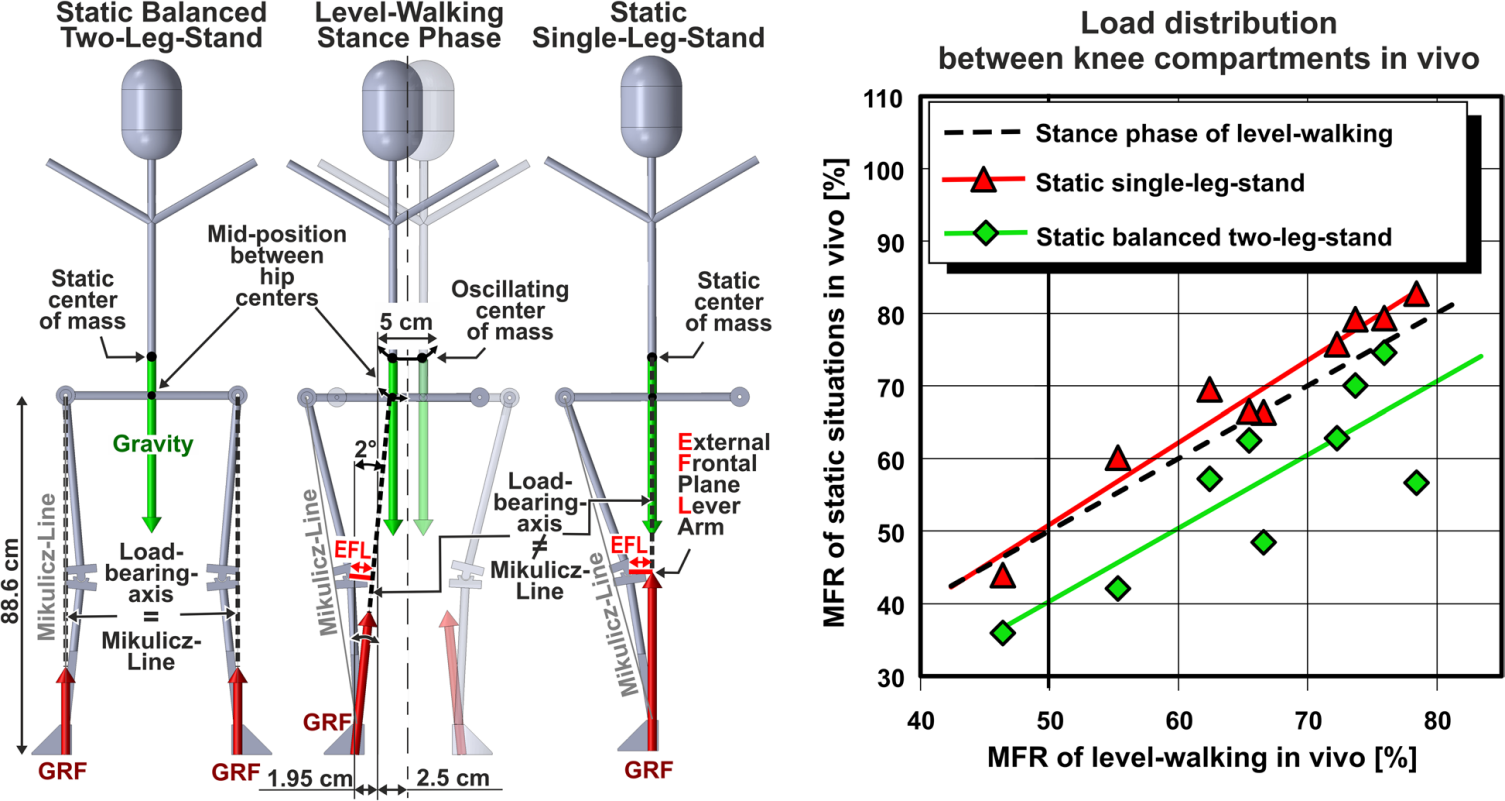
External and knee-internal frontal-plane knee load conditions for static and dynamic situations. Left: The frontal-plane projection of the variable Ground-Reaction-Force (GRF) acting towards the distal end of the tibia over the complete stance-phase of level-walking resembles, regarding its orientation and magnitude in weighted temporal average, much more the GRF of the static single-leg-stand than the GRF of the static balanced two-leg-stand. Similar to the GRF of the static single-leg-stand, the averaged level-walking-GRF approximately points towards the mid-position between both hip centres (Appendix A.1). The average dynamic load-bearing-axis of level-walking thus is markedly different from the Mikulicz-Line. As illustrated for a comfortable walking speed of 5 km/h and average leg length, the midpoint between both hip centres commutes quasi symmetrically around the intersection position of the hip-centre-to-hip-centre connection line with the temporally averaged load-bearing-axis of the predominantly loaded leg (illustration scaled by factor three in width for better visibility). Right: Correlations of the medial compartment force ratio (MFR) measured in vivo over the stance phase of level-walking (including single-leg- and two-leg-phases) with the MFR of the static single-leg-stand and the MFR of the static balanced two-leg-stand, both static situations analyzed over 1.9 s. A 50% MFR at static single-leg-stand involves a 50% MFR at level walking, too

With IFL*/w, KAM_S_, level-walking- and single-leg-stand-MFR being the independent variables, and EFL/w, KAM and the MFRs of the static two-leg- and single-leg-stand (Fig. 3 only) being the dependent ones, linear regression analysis was performed using Microsoft Excel 2019 (including Real Statistics Add-In). The coefficients of determination *R*^2^, the *P* values, statistical power and confidence intervals were thus identified. The statistical significance of the difference between two correlations was tested by the depending-overlapping-samples correlation t-test.

## Results

### Biomechanical findings

The frontal-plane load-bearing-axis of level-walking, averaged over the complete stance phase from heel strike to toe-off, was found virtually meeting the mid-position between both hip centres for comfortable walking speed. The load-bearing-axis of level-walking thus actually resembles much more the load-bearing-axis of the static single-leg-stand than that of the static balanced two-leg-stand (Fig. 3 left).

This external force orientation is reflected in the medio-lateral force distribution between the knee compartments in vivo: The medial compartment force ratio (MFR) over the stance phase of level-walking, which is the percentage of axial knee-internal force transferred to the medial compartment from heel strike to toe-off, was found only moderately correlated with the MFR of the static balanced two-leg-stand (*R*^2^ = 0.69, *P* = 0.006), but highly significantly correlated with the MFR of the static single-leg-stand (*R*^2^ = 0.95, *P* < 0.001). Despite the small sample size, the correlation of the level-walking-MFR was found significantly better (*P* = 0.037) with the MFR of the static single-leg-stand than with the MFR of the static balanced two-leg-stand. Due to the virtual identity especially near the 50% MFR (Fig. 3 right), balanced compartmental loads during static single-leg-stand will involve balanced compartmental loads during level-walking, too, which substantiates the appropriateness of our approach.

**Fig. 4:**
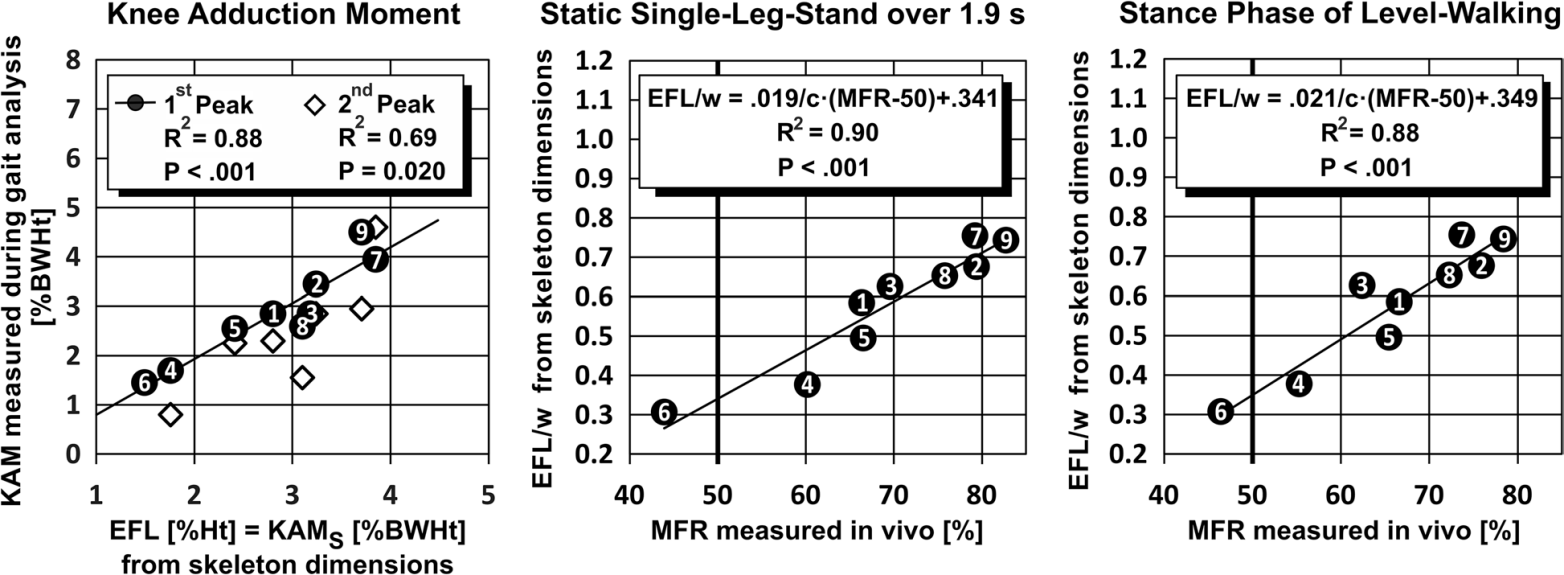
Relationships of the skeleton-derived EFL to KAM and MFR. Left: Relationships between the external Knee Adduction Moment at static single-leg-stand (KAM_S_), calculated from frontal-plane skeleton dimensions, and both published KAM peaks measured during gait analysis. Mid and right: Relationships between the Medial compartment Force Ratio (MFR) calculated from published in-vivo-measurement data, and the knee-width-normalized External Frontal plane Lever arm (EFL) calculated from frontal-plane skeleton dimensions (MFR-scale assuming c = 1.5). The respective relationships for the static single-leg-stand (mid) and for the stance phase of level-walking, including both single-support- and double-support-phases (right), are very similar. All data originate from the same nine subjects, the number within each mark corresponds to the number of the subject denotation

Moreover, the MFRs of the static single-leg-stand and the stance phase of level-walking in vivo are highly predictable by the external frontal-plane lever arm (EFL) calculated from frontal-plane skeleton dimensions. Linear regression analysis revealed highly significant positive correlations of the knee-width-normalized EFL/w with the knee-width-normalized knee-internal frontal plane lever arms IFL_SLS_/w and IFL_LW_/w calculated from in-vivo-measurement data, for the static single-leg-stand (intercept = 0.341; 68.3%CI, 0.304–0.379; 95%CI, 0.259–0.423; EFL/w = 1.85·IFL_SLS_/w + 0.341; t_7_ = 9.9; *P* < 0.001; *R*^2^ = 0.90) and for the stance phase of level-walking (intercept = 0.349; 68.3%CI, 0.308–0.391; 95%CI, 0.259–0.440; EFL/w = 2.12·IFL_LW_/w + 0.349; t_7_ = 9.1; *P* < 0.001; *R*^2^ = 0.88). IFL_SLS_/w and IFL_LW_/w are proportional to the respective medial compartment force ratio (MFR, Eq. (4)). The one EFL/w corresponding to MFR = 50% results from the intercepts of the latter two relationships to EFL/w = 0.341 for the static single-leg-stand, and, very similar, EFL/w = 0.349 for the stance phase of level-walking (Fig. 4 mid/right). Balanced loads during the stance phase of level-walking thus may be expected if the EFL is 0.349 times the femur epicondyle distance w, which is the sought-after EFL for osteotomy around the knee.

Furthermore, the skeleton-derived static knee adduction moment KAM_S_, equal to EFL in the unit [%Ht], turned out highly predictive for the published knee adduction moment (KAM) measured during gait analysis. Highly significant linear correlations of KAM with KAM_S_ were found for the maximum KAM (estimate: 68.3%CI; KAM = 1.26·KAM_S_-0.61%BWHt ± 0.44%BWHt; t_7_ = 7.1; *P* < 0.001; *R*^2^ = 0.88), and especially for the first KAM peak, values being even almost equal (Fig. 4 left) (estimate: 68.3%CI; KAM = 1.13·KAM_S_-0.33%BWHt ± 0.40%BWHt; t_7_ = 7.0; *P* < 0.001; *R*^2^ = 0.88).

For the given nine subjects, the published KAM measured during gait analysis is correlating better with the skeleton-derived EFL than with the MA for all peaks of the measured KAM (Table 1).

**Table 1:** Comparison between EFL and MA regarding their correlation with the measured KAM

	^a^KAM Maximum		^a^KAM 1^st^ Peak		^a,c^KAM 2^nd^ Peak	
	^b^EFL	MA	^b^EFL	MA	^b^EFL	MA
*R*^2^	0.88	0.84	0.88	0.87	0.69	0.61
*P* value	< 0.001	< 0.001	< 0.001	< 0.001	0.020	0.038

^a^Published KAM in [%BWHt]

^b^Skeleton-derived EFL in [%Ht]

^c^2^nd^ Peak exhibited by seven subjects only

The MFR as well is correlating better with this EFL than with the MA at static single-leg-stand, for the complete stance phase of level-walking, and even for the static balanced two-leg-stand (Table 2).

**Table 2:** Comparison between EFL and MA regarding their relationship to the MFR measured in vivo

	Static single-leg-stand		Level-walking								Static balanced two-leg-stand	
			100% Stance phase		^a^|M_Y_|≥ 63%BWcm 78% Stance phase		^a^|M_Y_|≥ 100%BWcm 67% Stance phase		^a^|M_Y_|≥ 140%BWcm 59% Stance phase			
	^b^EFL	MA	^b^EFL	MA	^b^EFL	MA	^b^EFL	MA	^b^EFL	MA	^b^EFL	MA
*R*^2^	0.90	0.83	0.88	0.86	0.85	0.80	0.77	0.69	0.68	0.58	0.631	0.617
*P* value	< 0.001	< 0.001	< 0.001	< 0.001	<0.001	0.001	0.002	0.006	0.006	0.016	0.011	0.012

^a^|M_Y_| is proportional to the force difference between the knee compartments (Appendix A.3)

^b^Skeleton-derived EFL normalized to femur epicondyle distance w

### Clinical relevance

**Fig. 5:**
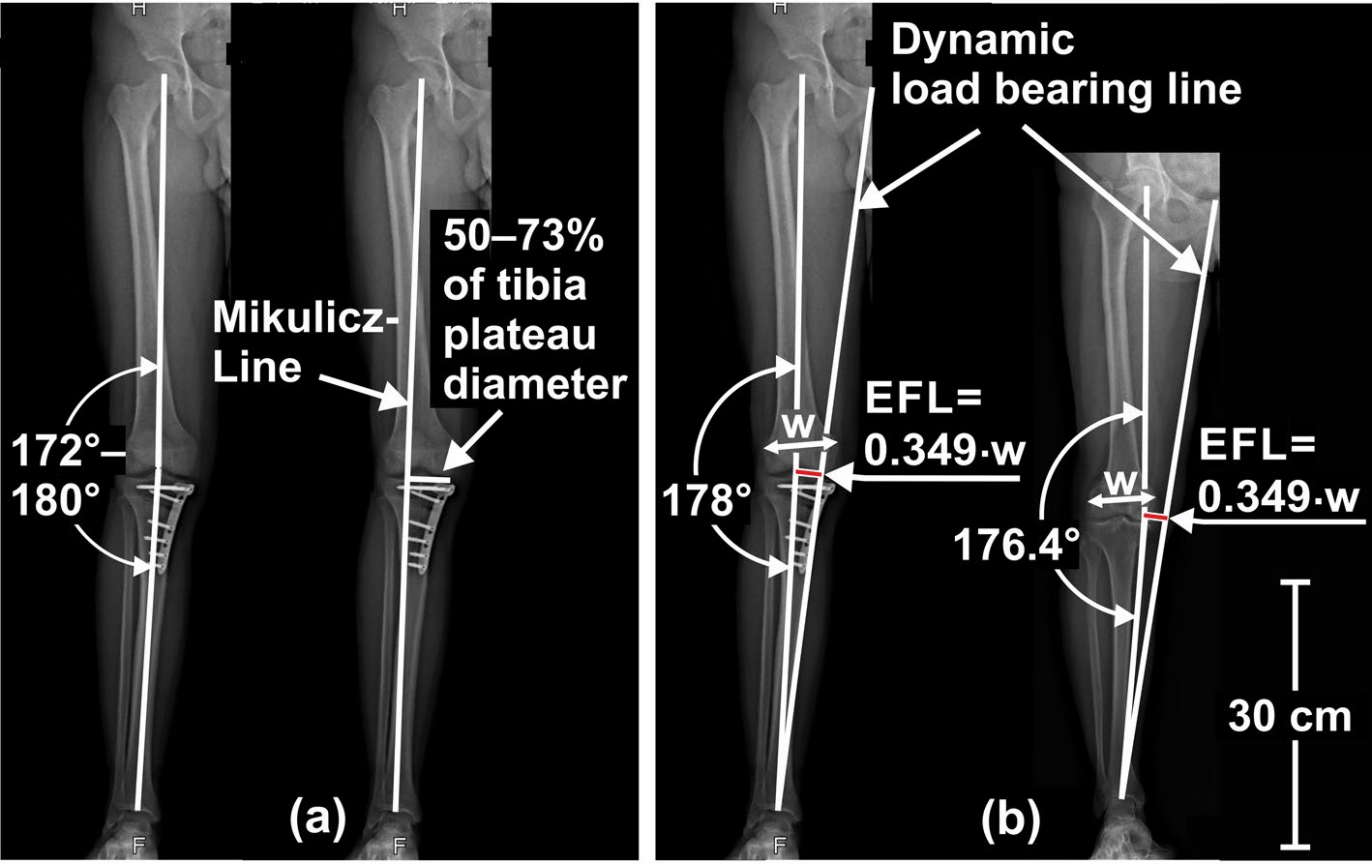
Proposal for the planning of osteotomy around the knee using EFL as target parameter. Hitherto planning of osteotomy around the knee targets either at a defined axis angle or at a defined distance of the Mikulicz-Line to the medial edge of the tibia plateau (**a**). We propose to target at a defined, knee-width-dependent distance from the knee centre to a dynamic load bearing line, which connects the talotibial joint centre with the mid-position between both hip centres, approximately given by the cranial end of the symphysis pubis (**b**). With this distance, denoted as EFL, variable target MAs result for the individually different distances of femur epicondyles, hip centres, femur- and tibia-lengths

**Fig. 6:**
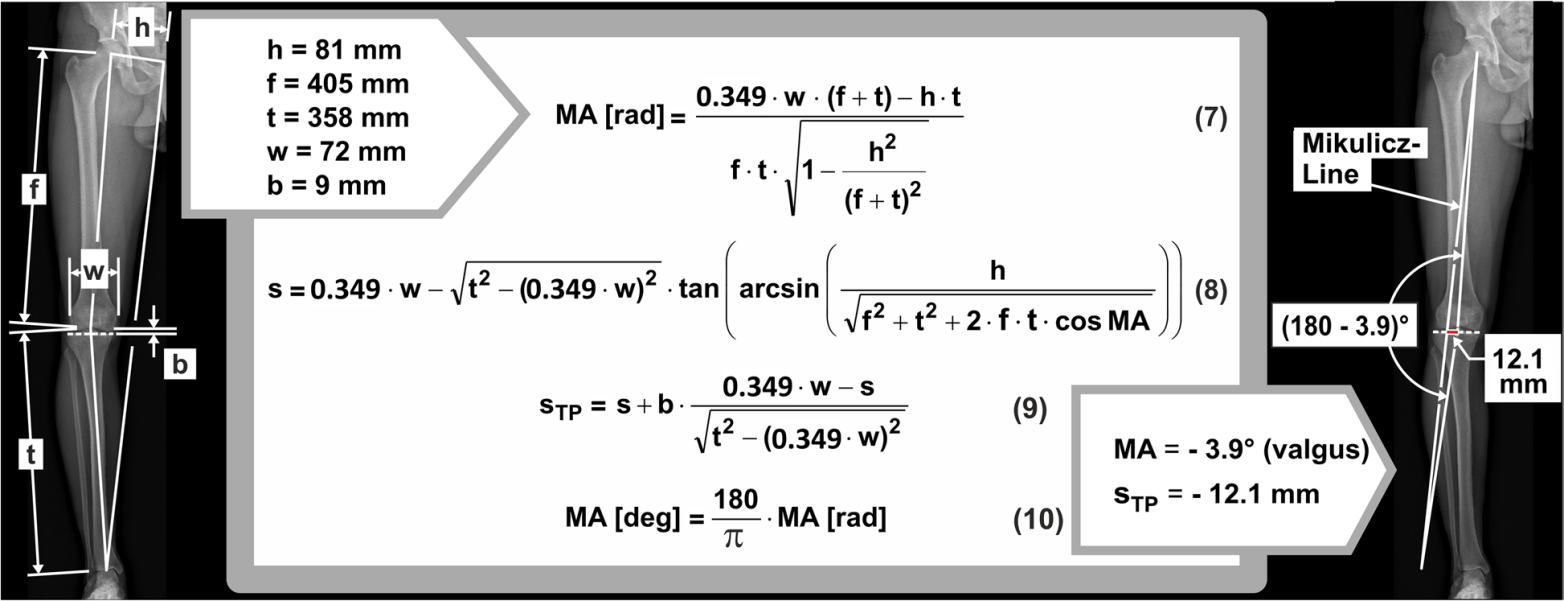
Formulas for the calculation of the MA and the Mikulicz-Line-position from skeleton dimensions. The exemplarily measured dimensions inserted into the formulas, which must be calculated successively in the order of their numbering while using results of preceding formulas, finally result in the target MA and the lateral distance s_TP_ of the Mikulicz-Line from the knee centre on the tibia plateau

As the EFL derived from frontal-plane skeleton dimensions is a physically more comprehensive and accurate predictor for the external frontal-plane knee torque, i.e. the KAM, during static and dynamic single-leg-situations than the MA, the KAM, again, is highly correlated with the medial compartment force ratio (MFR) over the complete stance phase of walking gait [42], EFL appears to be a more efficient target parameter to achieve balanced compartmental loads (MFR = 50%) by osteotomy around the knee than the MA. The target EFL should be 0.349 times the femur epicondyle distance w to achieve balanced compartmental pressure distribution during level-walking in temporal average (Fig. 5(b)). Detailed planning with the resulting formulas (7)-(10) is exemplified in Fig. 6. A calculator using these implemented formulas is provided with the Additional file 1.

### Evaluation of correction formulas regarding anthropometrically established body proportions

Results of the correction formulas (7)-(10) (Fig. 6) applied to fictive patients with realistic skeleton dimensions are compiled in Table 3: For average sized patients with average body proportions, an average correction recommendation of 3.4° valgus for the MA and an intersection of the Mikulicz-Line with the tibia plateau at 62% of the tibia plateau diameter, measured from the medial edge, results. For deviant skeleton geometries, different target MAs are required. Based on a confidence level of 68.3%, regarded as reasonable for physical measurements [23], the target correction intervals of the margin figures “lanky man” and “stocky women” are clearly separated from the target intervals of average figures. For a confidence level of 95%, the confidence interval of the target MA is enlarged to ± 2.1° about the mean. In any case, valgus alignment appears appropriate for average patients.

**Table 3:** Correction examples for fictive patients with realistic skeleton dimensions

Patient’s gender and figure	Body height, mm	h, mm	f, mm	t, mm	w, mm	b, mm	Target MA, °valgus^a^	Target position Mikulicz-Line on tibia plateau (%)^a^	Target position dynamic load-bearing-line on tibia plateau (%)^a^
*Male*									
Average	1790	90.8	461	380	84	11.8	3.3 ± 1.0	61.3 ± 4.2	14 ± 4.1
Broad-framed	1890	105	487	400	88	11.8	4.4 ± 1.0	66 ± 4.2	14 ± 4.1
Gracile	1690	72	435	360	79	11.8	1.4 ± 1.0	53 ± 4.2	14 ± 4.1
Lanky	1790	72	461	380	84	11.8	0.9 ± 1.0	51 ± 4.2	14 ± 4.1
*Female*									
Average	1660	87.8	432	349	79	11.3	3.4 ± 1.0	62.5 ± 4.4	12 ± 4.4
Broad-framed	1760	105	458	368	84	11.3	5.0 ± 1.0	69 ± 4.4	12 ± 4.4
Gracile	1560	70	405	329	75	11.3	1.7 ± 1.0	54 ± 4.4	12 ± 4.4
Stocky	1660	105	432	349	79	11.3	5.8 ± 1.0	73 ± 4.4	12 ± 4.4

^a^Error range calculated from the margins of the 68.3% Confidence Interval (CI) of intercept (mean ± 1.08 times standard error of intercept)

The first, mostly highest KAM peak from gait analysis would vary between 1.7 ± 0.4%BWHt for the “lanky man” and 2.9 ± 0.4%BWHt for the “stocky woman”, if all fictive patients were aligned to zero MA. The KAM of the neutrally aligned “stocky woman” would correspond to the KAM of the “lanky man”, if the latter was 5° varus aligned. Both patients might suffer from similar medial knee overload and profit from osteotomy around the knee, however with considerably different target angles.

## Discussion

The developed correction formulas propose a method for quantitative planning of osteotomy around the knee including a crucial adduction moment parameter. Based on individual skeleton dimensions, which can be read from standard full-leg-radiographs, patient-specific target angles and Mikulicz-Line positions can be calculated.

Results for average patients, requiring a target MA of 3.4° valgus and a target position of the Mikulicz-Line at 62% of the tibia plateau diameter, exactly confirm the most consented correction recommendation for varus precondition: 3–5° valgus [53] and a Mikulicz-Line position at 62–62.5% of the tibia plateau diameter (“Fujisawa-Point”) [45, 60]. Results for average patients suggest that the established correction recommendations already account for the adduction moment by moderately overcorrecting into valgus alignment. This overcorrection does not lead to an overload of the lateral knee compartment, as generally assumed, but is mandatory to guarantee balanced compartmental pressures with regard to the highly load-bearing dynamic gait situation.

For skeleton proportions deviating from average, aberrant target MAs are needed, possibly an explanation for the large variability of correction recommendations between mostly 0° for valgus precondition [52] and 3–8° valgus for varus precondition [60]. The inconclusiveness with regard to the optimal degree of (over)correction might be explained by skeleton differences between and within the respective patient populations, requiring very variable target angles and thus precluding commitment to one value, as every patient is equipped with highly individualized anatomic parameters. Our results for fictive, but realistic patients (target MA 0.9–5.8° valgus, Mikulicz-Line position at 51–73% of the tibia plateau diameter), are consistent with empirical findings concerning favourable long-term outcome, for the intersection position of the Mikulicz-Line with the tibia plateau (50–73%) [16, 20] as well as for the target MA of leg alignment (0–8°valgus) for varus and for valgus precondition [33, 52, 60]. Hernigou et al. (1987) [33] for example, frequently cited to justify close-to-neutral re-alignment below 3° valgus (e.g. [15, 47]), factually found degeneration of the lateral compartment due to overcorrection not before 7–10° valgus, some good results between 0–3° valgus, but optimal clinical and radiographical ten-year-outcome at varus precondition for a postoperative MA between 3–6° valgus. Our correction examples (Table 3) meet the favourable range.

Concordant to our results for average patients, up-to-date computational studies, combining 3D-magnetic-resonance data with finite-element-analysis and including the adduction moment from gait analysis, likewise found balanced compartmental stress for a Mikulicz-Line position at 60–65% of the tibia plateau diameter [47] and (averaged) 3.6° valgus alignment [70].

For average patients with varus precondition, our results confirm rather the established consensus to position the Mikulicz-Line to the Fujisawa-Point than the recent trend for close-to-neutral re-alignment. The apparently only one publication reporting on a better (mid-term) outcome for mild correction, optimal for a Mikulicz-Line position at 50–55% of the tibia plateau diameter from medial [36], compares the outcome of patients, whose planned degree of correction had been preoperatively assigned to the degree of knee damage, a higher degree of correction assigned to more damaged knees. As osteotomy prognosis worsens with preoperative damage [61], the worse outcome for higher correction angles might be self-explanatory.

The recent trend towards neutral re-alignment may be partially ascribed to static load tests using isolated cadaveric knees [1, 48], which found balanced compartmental pressures when the load-bearing-axis intersected the tibia plateau within the medial compartment and the MA was varus-aligned (Appendix A.2). This led to the conclusion that overcorrection into valgus alignment might not be necessary. However, shifting the Mikulicz-Line to this equilibrium position in the medial compartment would equalize compartmental pressures only for the static balanced two-leg-stand (Fig. 3), thus completely disregarding highly load-bearing (dynamic) single-support situations with load-bearing-axes much more medial than the Mikulicz-Line. The coming into effect of different weight-bearing-lines at two-leg- compared to single-leg-situations, which is the basic concept of our theoretical approach, has already been recognized and amplified by Shaw et al. (1996) [59], and could be clearly confirmed by recent in vivo measurements: For all two-leg situations, slightly more load is distributed to the lateral compartment, whereas for all single-leg activities the medial compartment has to bear much higher loads [43]. Osteotomy planning according to our proposal will position the average dynamic load-bearing-axis of level-walking (Fig. 5) to 12–14% of the tibia plateau diameter measured from the medial edge (Table 3), exactly to the balanced-load-position (0–25%) found in the experiments with cadaveric knees [3]. The Mikulicz-Line, however, still passes the lateral compartment.

Another finding that might have fostered the concern about overcorrection to valgus alignment is the dramatic increase of total joint contact forces with increasing valgus alignment computed in earlier studies [30, 31]. It led to recommendations for neutral realignment at varus precondition. A very recent study, however, co-authored partly by the same authors, proved these concerns unnecessary, as it found the total joint contact forces not significantly influenced by varus/valgus malalignment in vivo [67]. Further studies thoroughly ferreted out tiniest degenerative changes in the lateral compartment, with the alarming conclusion that even slight valgus alignment might be detrimental [14, 22]. Investigations, however, were restricted to changes in the lateral compartment alone, thus completely disregarding possibly even worse degradation for neutral alignment within the medial compartment. Studies including both compartments, originating from knees of partly the same cohort (“MOST”), found that valgus knees, in the same period, had a clearly reduced risk versus neutral alignment for incident distinct radiographic osteoarthritis including osteophytes [57], and valgus alignment, regardless of its degree, did not increase the risk of incident lateral, but progressively decreased the risk of incident medial cartilage damage compared to neutral alignment [58].

Despite reasonably balanced varus-to-valgus prevalence [7], isolated gonarthrosis was observed fourteen times more in the medial than in the lateral compartment [32], and osteoarthritic individuals are more likely to have neutral than valgus alignment compared to healthy ones [68]. Evidence suggests that slight valgus alignment might be even healthier than neutral alignment with regard to knee survival until old age, and the recent trend for close-to-neutral realignment in case of medial compartment damage should be reconsidered, as it might be based on a misconception.

The biomechanical findings of this study, to our knowledge unprecedented so far, suggest that the KAM is influenced not only by the MA, but also by the distance between both hip centres, the femur- and tibia-length. The extent of relationship between KAM and MA thus depends on the variability of these additional skeleton dimensions compared to the variability of the MA in the respective patient population. This might explain, at least in parts, the merely moderate and extremely variable correlation of the maximal KAM with the MA in former studies [37, 51, 62] and the poor relationship between changes of the MA and changes of the KAM by osteotomy around the knee in various patient populations [40].

Our numerical results are clearly limited by their partial dependence on load measurements in non-physiologic knees after arthroplasty, even if the subjects had regained normal gait at the time of data acquisition. The cruciate ligaments had been sacrificed, thus forces normally taken up by these ligaments are transferred to the implant [13]. As the cruciate ligaments are acting predominantly parallel to the tibial plateau, the axial forces relevant for our results might be less affected. Another limitation is the small number of available subjects with instrumented prostheses. Statistical power for simple linear regression, however, is sufficient (e.g. 0.83 for α = 0.001, *R*^2^ = 0.84, 9 samples), and after all, the identified linear correlations are (highly) significant. Amazingly, the necessary sample size for linear regression is still under discussion and was found surprisingly small [6, 27, 39], possibly because statistical efficiency of simple linear regression depends not only on sample size, but also on range and distribution of the independent variable values [27]. For simple linear regression, three variable pairs selectively chosen at the beginning, mid and end of the possible range may statistically outperform even larger numbers of randomly chosen pairs agglomerating near the centre of that range. Referring to this, the subject population under study is fortuitously well-composed. MAs range from 4.5° valgus to 7° varus, which covers 88.5% of the MA-range of a randomly chosen population with *n* = 500 [7]. This might explain the highly significant linear regression results despite moderate sample size. Concerning the correlation t-test, however, sample size is too small to underpin the observed small differences in the coefficients of determination given in Tables 1 and 2 with sufficient statistical significance. These quite small differences might be due to the fact that the subjects under study are very homogeneous regarding half hip-centre-to-to-hip-centre distance (88.4 ± 4.1 mm) and body height (1720 ± 38 mm), whereas their MA differs considerably (2.39 ± 3.94°) compared to representative larger populations [2, 7, 29]. The subjects’ MA thus explains almost all variance of their skeleton-derived EFL (*R*^2^ = 0.97), which is reflected in the, compared to literature, extraordinarily high correlation of the subjects’ maximal KAM with their MA (*R* = *r* = 0.92 versus *r* = 0.45 [62], *r* = 0.703 [51], *r* = 0.14–0.79 [37]. Still better correlation of KAM and MFR with the EFL than with the MA would be expectable for collectives with higher skeleton- and lower MA-variability, which might be subject of further research.

Finally, formulas have been evaluated using fictive patients only. Future research might retrospectively relate known outcome of real osteotomy patients to the deviation of achieved re-alignment from the proposed target angle.

## Conclusion

The external frontal plane lever arm (EFL) of the knee adduction moment, essentially derivable from a full-leg radiograph with depicted symphysis pubis, is a more appropriate target parameter for planning osteotomy around the knee than the angle of static leg alignment. Starting from an EFL which is proportional to the femur epicondyle distance, individual target angles can be calculated from the femur- and tibia-length and the distance from the hip centre to the symphysis pubis, all dimensions read from a frontal-plane full-leg radiograph. The calculated values provide a guideline, which may be varied as appropriate. More correction might be adequate to further unload a severely damaged compartment, less correction for merely preventive realignments in young patients who wish to remain further engaged in competitive sports.

The proposed method enables a practicable quantitative inclusion of a crucial adduction moment parameter into planning of osteotomy around the knee. Using the correction formulas (7)-(10) (Fig. 6), automatized in the Additional file 1, the method can be implemented with immediate effect, and improved outcome might be expected.

## Supplementary Information

**Additional file 1.** Calculator for Formulas (7)-(10). Excel-spreadsheet to calculate the target angle for osteotomy from skeleton dimensions according to Fig. 6.

**Additional file 2.** Influence of the tibio-femoral alignment on compartmental contact-areas and -pressures. Figure supplementing Appendix A.2.

**Additional file 3.** Derivation of the total bony leg length from body height. Figure supplementing Appendix A.6.

## Data Availability

The datasets supporting the conclusions of this article are included within the article and its additional files provided in the database OrthoLoad [11, 12]. This database allows, for non-commercial applications, to use videos, screenshots of the videos or numerical data as long as the database www.OrthoLoad.com and the source file name is referenced. Using any content from OrthoLoad.com in a commercial application requires explicit permission of the OrthoLoad editors.

## Appendix

### A.1 Position of the dynamic load-bearing-axis of level-walking

During level-walking, the “body **C**entre **o**f **M**ass (**CoM**)” is periodically moving [64] and the ground-reaction-force (GRF) is periodically changing its magnitude and direction [49], both dependent on gait velocity. In contrast to static single-leg-stand, the CoM is almost never perpendicular above the base of support during the dynamic single-leg phase of level-walking [35], but is travelling medial and very close to the base of support of the predominantly loaded leg over midstance [46]. Its closest distance from the base-of-support centroid at midstance, when lateral displacement of the CoM is highest [50], is (averaged) 1.95 cm for a self-selected walking speed of 1.38 m/s in healthy young adults [46]. The peak-to-peak lateral displacement of the CoM, again, is restricted to 5 cm (± 2.5 cm from its central position at load transfer between both legs), for a comfortable, energy-efficient walking speed of 1.4 m/s (5 km/h) [64]. Thus, related to the predominantly loaded leg, the CoM is varying its medio-lateral distance from the base-of-support centroid from 1.95 cm to (1.95 + 2.5) cm (Fig. 3), average distance thus being 3.2 cm. As the frontal-plane CoM position can be approximately located at mid pelvis without significant lateral movement error during gait for healthy subjects [19], and the average pelvic tilt of (averaged) 0.15° over 20–80% of the stance phase [44] can be neglected, the CoM can be assumed, in temporal average, perpendicular above the midpoint of the hip-centre-to-hip-centre connection line. Hence, the medio-lateral distance of this midpoint to the base-of-support centroid is 3.2 cm in temporal average, too.

Projected to the frontal-plane, the average weighted tilt of the GRF towards vertical results to 2.0° from the arc tangent of the proportion between lateral and vertical ground-reaction-impulse over the stance phase of level-walking, which is about 15.2 Ns and 445 Ns, respectively, for a walking speed of 1.4 m/s [49]. With an average leg length from the hip centre to the ground of 88.6 cm (half average German body height, which is 172.5 cm [63] corresponding to the height of the symphysis pubis [54], the latter approximately at height of the distal rim of the femoral head, plus half average diameter of the femoral head, which is 4.77 cm [2]), the tilted average frontal-plane GRF axis (dynamic load-bearing-axis) meets the connection line between both hip centres about 3.1 cm medial from the base-of-support centroid position of the predominantly loaded leg (average pelvic tilt neglectable, again). The dynamic load-bearing-axis thus meets the hip-centre-to-hip-centre connection line 3.1 cm minus 3.2 cm distant from its average mid-position, thus meeting the mid-position between both hip centres quite exactly in temporal average (Fig. 3).

The average GRF magnitude for a walking speed of 1.4 m/s, derived from the findings of Nilsson and Thorstensson (1989) [49], results from the ratio of the frontal-plane ground-reaction-impulse-vector magnitude (445.3 Ns) and the support time (0.72 s) to 618 N, which corresponds to 82% of the average body weight (77 kg for the subject population under examination [49]). This force is illustrated by the length of the GRF arrow for level-walking in Fig. 3. The GRF magnitude per leg for the static balanced two-leg-stand is about 50%, for static single-leg-stand about 100% of body weight.

### A.2 Medial compartment force ratio (MFR) required for equal compartmental pressure distribution

We exploited publications reporting on compartmental pressure- and contact-area-measurements in isolated cadaveric knees using TekScan electronic sensor technology, which came up with the new millennium. Compared to the formerly popular ink-based pressure sensitive film technology (e.g. Fuji film), the lower thickness of the TekScan sensor film allows better adaptation to ball-shaped contact surfaces, and accuracy and reproducibility are much higher [28, 34]. The former tended to severely underestimate the contact area at lower pressures and delivered unsatisfactory results especially for congruent joints [28], what might have contributed to the conclusion that the lateral knee compartment is less congruent than the medial one [3].

Most of the four identified publications measured average pressures and areas over a larger range of alignments and thus force distributions. After linearly interpolating the measured average pressure in the medial and lateral compartment within the one range, where the difference between the average medial and lateral pressure changed sign, we found the alignment necessary for equal pressure distribution at the intersection of the alignment-pressure-function for the medial compartment with the alignment-pressure-function for the lateral compartment.

The force in the medial and lateral compartment (and thus force distribution) resulted from the product of equilibrium pressure and respective medial and lateral contact area, the latter determined by linear interpolation of average medial and lateral contact areas within the same alignment range (illustrated in the Additional file 2).

Balanced average compartmental pressure was found for the axial knee force distributed as follows:

- Forty-seven percent to the medial and 53% to the lateral compartment when the load axis crossed the tibiofemoral joint line at 17% of its length from medial in 6 knees [1];
- Forty-two percent to the medial and 58% to the lateral compartment for a tibiofemoral angle of -2° (valgus) in 8 knees with intact cartilage [48];
- Fifty-one percent to the medial and 49% to the lateral compartment for an anatomical angle of -2.8° (valgus) in 5 knees [52];
- Seventy percent to the medial and 30% to the lateral compartment for a tibiofemoral angle of 2° (varus) in 4 knees [65]. This author applied only this one force distribution, which led to reasonably balanced contact stress (8 ± 2.5 MPa in the medial and 7.6 ± 2.3 MPa in the lateral compartment).

Averaged results of these four publications, weighted by the respective number of knees under investigation, resulted in an axial force distribution between the medial and lateral compartment of 50% for a total of 23 knees. Equal average pressure in both knee compartments thus can be expected if the MFR is 50%.

All four publications agree in the finding that balanced compartmental pressures will be achieved if the load axis passes the medial compartment and the leg’s mechanical axis is varus-aligned, as the identified tibiofemoral (anatomical) valgus angles correspond to MAs, which are about 5–6° more varus [24].

### A.3 Derivation of the MFR for a defined period of time

The force acting within the medial/lateral knee compartment in cranio-caudal direction, F_med_ and F_lat_, respectively, is given by [43]

11 $$\begin{array}{*{20}c} {{\text{F}}_{{{\text{med}}}} = \frac{1}{2} \cdot \left( { - {\text{F}}_{{\text{Z}}} } \right) - \frac{{{\text{M}}_{{\text{Y}}} }}{{l}}} & {{\text{F}}_{{{\text{lat}}}} = \frac{1}{2} \cdot \left( { - {\text{F}}_{{\text{Z}}} } \right) + \frac{{{\text{M}}_{{\text{Y}}} }}{{l}}} \\ \end{array}$$

with (-F_Z_) being the knee-internal force component along the tibia axis in cranio-caudal direction, (-M_Y_) being the knee-internal torque in the frontal-plane, which shifts surplus load to the medial compartment and unloads the lateral one by the same amount, and l being the femur condyle distance at the distal femur end, assumed to be proportional to the epicondyle distance w (c: proportionality constant):

12 $$l = \frac{{\text{w}}}{{\text{c}}}$$

The axial force difference between the knee compartments thus is proportional to the frontal-plane torque -M_Y_:

13 $${\text{F}}_{{{\text{med}}}} - {\text{F}}_{{{\text{lat}}}} = - 2 \cdot \frac{{{\text{M}}_{{\text{Y}}} }}{l}$$

The MFR, with Eq. (11), is given by

14 $$\begin{array}{*{20}c} {{\text{MFR}} = \frac{{{\text{F}}_{{{\text{med}}}} }}{{\left( { - {\text{F}}_{{\text{Z}}} } \right)}} \cdot 100} & { = \left( {\frac{1}{2} + \frac{{{\text{M}}_{{\text{Y}}} }}{{{\text{F}}_{{\text{Z}}} \cdot l}}} \right) \cdot 100} \\ \end{array}$$

For each instant, M_Y_ can be represented as the product of F_Z_, composed from muscle forces and gravity, and a knee-Internal Frontal plane Lever arm (IFL), which can be conceived as the distance of the action line of F_Z_ from the knee centre in the frontal-plane:

15 $${\text{M}}_{{\text{Y}}} = {\text{IFL}} \cdot {\text{F}}_{{\text{Z}}}$$

Inserted in (14) with (12):

16 $${\text{MFR = }}\left( {\frac{1}{2} + {\text{c}} \cdot \frac{{{\text{IFL}}}}{{\text{w}}}} \right) \cdot 100$$

Hence, an MFR of 50% will be achieved if IFL/w equals zero.

During the load cycle of gait, knee-internal forces and torques are changing, alternately shifting surplus load to the medial or lateral compartment [13]. Equal load distribution cannot be achieved for each instant, but for i instants equally distributed over an investigated period, the respective torques M_Yi_, which are proportional to the force differences between the knee-compartments (Eq. (13)), must sum up to zero for balanced compartmental forces during this period:

17 $$\sum\limits_{{\text{i}}} {{\text{M}}_{{{\text{Yi}}}} = 0}$$

Hence, for all IFLs occurring at these instants, denoted as IFL_i_, with (15) the following relation must apply:

18 $$\sum\limits_{{\text{i}}} {{\text{IFL}}_{{\text{i}}} \cdot {\text{F}}_{{{\text{Zi}}}} = 0}$$

There is one special IFL in-between these IFL_i_, denoted as IFL* below, which, comparable to a gravity centre, would leave the sum of torques unchanged, if all forces F_Zi_, occurring at any instant during the investigated period, acted only in this distance IFL* from the knee centre:

19 $${\text{IFL*}} \cdot \sum\limits_{{\text{i}}} {{\text{F}}_{{{\text{Zi}}}} = } \sum\limits_{{\text{i}}} {{\text{IFL}}_{{\text{i}}} \cdot {\text{F}}_{{{\text{Zi}}}} }$$

20 $$\iff \text{IFL*} =\frac{\sum \limits_{\mathrm{i}}{\mathrm{M}}_{\mathrm{Yi}}}{\sum \limits_{\mathrm{i}}{\mathrm{F}}_{\mathrm{Zi}}}$$

Inserting IFL* into Eq. (16) delivers the MFR for the investigated period

21 $${\text{MFR}} = \left( {\frac{1}{2} + {\text{c}} \cdot \frac{{\text{IFL*}}}{{\text{w}}}} \right) \cdot 100$$

A fifty-percent-MFR, with loads equally distributed over the investigated period, thus will be achieved if IFL*/w equals zero.

### A.4 Origin of the in-vivo-measurement data

The in-vivo-measurement data from the nine subjects with instrumented knee prostheses originate from the online database OrthoLoad [12, 9].

Static single-leg-stand data files [9]:

**Table:**

K1L_281008_1_18p.akf	K1L_281008_1_15p.akf	K1L_110108_1_95p.akf	K1L_110108_1_94p.akf
K2L_240311_1_18p.akf	K2L_240311_1_17p.akf	K2L_290409_1_23p.akf	K2L_290409_1_25p.akf
K2L_290409_1_26p.akf	K3R_291008_1_12p.akf	K3R_291008_1_14p.akf	K3R_090609_2_71p.akf
K3R_090609_2_63p.akf	K3R_090609_2_65p.akf	K4R_200312_1_32p.akf	K5R_230311_1_17p.akf
K5R_230311_1_19p.akf	K5R_060809_1_36p.akf	K5R_060809_1_43p.akf	K5R_060809_1_34p.akf
K5R_280415_1_71p.akf	K6L_210311_1_17p.akf	K6L_210311_1_14p.akf	K7L_280710_1_18p.akf
K7L_280710_1_21p.akf	K7L_270515_1_82p.akf	K8L_250311_1_15p.akf	K8L_250311_1_14p.akf
K8L_280415_1_24p.akf	K9L_290710_1_25p.akf	K9L_290710_1_22p.akf	

Static balanced two-leg-stand data files [9]:

**Table:**

K1L_240309_2_159p.akf	K2L_290409_1_21p.akf	K3R_250309_2_119p.akf	K4R_161009_1_35p.akf
K5R_260210_1_27p.akf	K6L_210311_1_46p.akf	K7L_121109_1_102p.akf	K8L_250310_1_86p.akf
K9L_050510_1_15p.akf			

Level-walking data files [8, 9]:

**Table:**

K1L_Walking.xlsx	K2L_Walking.xlsx	K3R_Walking.xlsx	K4R_200312_1_43p.akf
K5R_Walking.xlsx	K6L_Walking.xlsx	K7L_Walking.xlsx	K8L_Walking.xlsx
K9L_Walking.xlsx			

The *_Walking.xlsx files contain pre-processed, averaged level-walking data represented over one load cycle of gait for eight of the nine subjects. For the remaining subject K4R, the available level-walking raw data (K4R_200312_1_43p.akf) [9], comprising several load cycles of gait, were averaged to one resulting load cycle with the same method [13] used for all other subjects. The resulting file k4r_2242_0_7_i1.akf is available as supplemental file at the OrthoLoad database [11].

### A.5 Calculation of the subjects’ various IFL*, EFL and KAM_s_ values from raw data

From the level-walking data files containing pre-processed, averaged level-walking data represented over one load cycle of gait, the F_Zi_ and M_Yi_ values occurring over predefined periods of the load cycle, inserted in Eq. (5), delivered various subject-specific IFL*:

Calculated for the complete stance phase of gait, the relevant period to be influenced by osteotomy, IFL* is denoted as IFL_LW_ below. The stance phase was demarcated by comparing the total tibio-femoral contact force (F_RES_) to the subjects’ individual body weight (BW), both given in above data files. On average, F_RES_ is 85%BW at heel-strike of the ipsilateral foot and 48%BW at its toe-off [18].

IFL* was further calculated for several sub-periods of the stance phase characterized by

- Moderate (|M_Y_|≥ 63%BWcm),
- High (|M_Y_|≥ 100%BWcm),
- and highest (|M_Y_|≥ 140%BWcm)

knee-internal frontal-plane torque magnitudes, which are proportional to the compartmental force differences (Appendix A.3).

From the files containing static single-leg-stand data, M_Yi_ and F_Zi_ were read out for each subject for the first 1.9 s of the single-support phase, the maximal period covered by all trial data. Single-leg-stand data readout started with the instant of the first total force (F) maximum on the single-leg-support plateau. From the files containing static balanced two-leg-stand data, M_Yi_ and F_Zi_ were read out for each subject for the first 1.9 s from the beginning of the measurement. These M_Yi_ and F_Zi_ values inserted in Eq. (5) delivered the individual IFL*-values for the static single-leg-stand, denoted as IFL_SLS_ below, and for the static balanced two-leg-stand, denoted as IFL_TLS_ below. In case of several files for the same subject, results were arithmetically averaged.

The subjects’ skeleton data were available as 3D-computed-tomography position vectors of both hip centres, both ipsilateral femur epicondyles and the ipsilateral medial malleolus [11]. After mathematically projecting both femur epicondyle positions to the plane spanned by the left and right hip centre and the medial malleolus, the subject-specific femur epicondyle distance w was calculated as the distance between these two projections. Half the distance between both hip centres delivered h. The approximate femur length resulted from the distance between the ipsilateral hip centre and the medial epicondyle projection, and the approximate tibia length from the distance between the medial epicondyle projection and the medial malleolus. To account for the cranio-caudal distance between the medial femur epicondyle and the knee centre, 28.2 and 25.3 mm for male and female subjects, respectively, were added to the approximate femur length and subtracted from the approximate tibia length. The thus resulting femur length corresponds to f. The thus resulting tibia length was prolonged by 1.1 mm to get t in order to account for the cartilage thickness covering the distal tibia [5], as the medial malleolus is approximately on a level with the centre of the talotibial joint junction of the distal tibia. The 28.2 and 25.3 mm distance between medial epicondyle and knee centre mentioned above may be justified as follows: The average distance between the medial epicondylar sulcus and the medial joint line in the coronal plane has been measured to average 29.2 mm for men and 26.3 mm for women [26]. The knee centre of rotation for the subjects with instrumented prostheses was assumed 1 mm proximal to the medial joint line, as cartilage and eminentia intercondylaris had been removed for implantation of the artificial knee joints, and only a small elevation is shaped at the centre of the artificial tibia plateau. The tip of this elevation with an estimated hight of 1 mm was presumed to be the knee centre of rotation.

h, f, t and the subject-specific MA [42] inserted in Eq. (1) Fig. 2 finally delivered the subject-specific EFL.

With GRF at static single-leg-stand being approximately 1·BW, the individual knee adduction moment at static single-leg-stand (KAM_S_), in the usual unit [%BWHt], resulted from the subject-specific EFL and the subject's body height (Ht) [42] as follows:

22 $$\begin{aligned}{{\text{KAM}}_{{\text{S}}} } & { = {{{\text{EFL}} \cdot {1} \cdot {\text{BW}}} \mathord{\left/ {\vphantom {{{\text{EFL}} \cdot {1} \cdot {\text{BW}}} {\left( {{\text{BW}} \cdot {\text{Ht}}} \right) \cdot {1}00}}} \right. \kern-\nulldelimiterspace} {\left( {{\text{BW}} \cdot {\text{Ht}}} \right) \cdot {1}00}}} \\ {} & {{{ = {\text{EFL}}} \mathord{\left/ {\vphantom {{ = {\text{EFL}}} {{\text{Ht}} \cdot {1}00\quad \quad \;\;\;\;\;\;\;\;\;\;\;\;\;\;}}} \right. \kern-\nulldelimiterspace} {{\text{Ht}} \cdot {1}00\quad \quad \;\;\;\;\;\;\;\;\;\;\;\;\;\;}}} \end{aligned}$$

Resulting subject-specific data are compiled in Table 4.

**Table 4:** Calculation results for the subjects with instrumented knee prostheses

^a^Subject (male/female)	^a^Ht, mm	^a^MA, °varus	h, mm	f, mm	t, mm	w, mm	EFL, mm	IFL_TLS_, mm	IFL_SLS_, mm	IFL_LW_, mm	KAM_S_, %BWHt
K1L(m)	1770	3.0	90	467	358	85	49.6	-0.8	9.3	9.4	2.80
K2L(m)	1710	5.0	81	431	380	82	55.5	13.5	16.1	14.2	3.24
K3R(m)	1750	3.5	89	444	413	89	55.6	4.3	11.6	7.3	3.18
K4R(f)	1700	-4.5	96	410	358	79	29.9	-4.1	5.4	2.8	1.76
K5R(m)	1750	1.0	84	449	383	85	42.2	7.1	9.4	8.8	2.41
K6L(f)	1740	-4.0	90	477	405	85	26.0	-7.9	-3.4	-2.0	1.49
K7L(f)	1660	6.5	92	423	358	85	63.9	11.3	16.5	13.4	3.85
K8L(m)	1740	4.0	88	452	373	83	54.0	7.1	14.2	12.3	3.10
K9L(m)	1660	7.0	87	434	343	83	61.5	3.7	18.1	15.7	3.71

^a^[42]

IFL_LW_, IFL_SLS_, IFL_TLS_ and EFL were divided by the subject-specific epicondyle distance w for knee-width-normalization. Inserting the resulting individual values in Eq. (4) (with arbitrary, but consistent c) delivered subject-specific MFRs for the static single-leg-stand, the static balanced two-leg-stand and for the stance phase of level-walking as well as for the sub-periods of the stance phase defined above.

### A.6 Calculation of skeleton dimensions for fictive patients with realistic skeleton geometries

On average, the distance from the symphysis pubis (approximately on a level with the caudal rim of the femoral head) to the ground is half of body height [54]. The average foot height is 4.4 and 4.3% of the body height for men and women, respectively [25]. The total bony leg length, given by the sum of the total bony femur- and bony tibia-length (both without cartilage), on average is 2.31 and 2.33 times the bony tibia length for men and women, respectively [24], with the total bony femur length measured from the proximal rim of the femoral head to the distal end of the medial femur condyle, and the bony tibia length measured from the tip of the eminentia intercondylaris to the centre of the talotibial joint junction. Cartilage layers were considered at the proximal/distal tibia end and at the distal femur end. The knee centre was assumed at the midpoint of the connecting line between the ceiling of the intercondylar notch and the tuberculum mediale of the eminentia intercondylaris. Using these elements of knowledge and further average dimensions and proportions from literature listed below, from any given body height (Ht) the “**T**otal bony **L**eg length (**TL**)” (illustrated in the Additional file 3), the “distance **KC** of the **K**nee **C**entre to the intercondylar notch and to the tuberculum mediale of the eminentia intercondylaris”, as well as f, t, b, w and the “diameter of the **T**ibia **P**lateau (**TP**)” can be calculated. With TP, and with s_TP_ resulting from Eq. (9) (Fig. 6), the “target **P**osition of the **M**ikulicz-Line on the tibia plateau in percent (**PM**)” can be computed. TP together with w and EFL/w = 0.349 delivers the “target **P**osition of the **D**ynamic load-bearing-line on the tibia plateau in percent (**PD**)” Both positions are measured from the medial edge of the tibia plateau.

Resultant formulas for respective entries in Table 3 are listed below:

**Table:**

TL	=	Ht/2-pfh·Ht + FH-CF-CTP-CDT + ET	23
KC	=	(CTPM + CF + NMC-ET)/2	24
t	=	TL/plt + KC + CDT	25
f	=	TL·(1–1/plt)-FH/2-NMC + KC	26
w	=	TL·(1–1/plt)/pfe	27
b	=	ET + KC	28
TP	=	TL/(plt·ptd)	29
PM	=	100·(TP/2-TCK-s_TP_)/TP	30
PD	=	100·(TP/2-TCK-0.349·w)/TP	31

Therein, the following denotations, data sources and data were used:

pfh: Average **p**roportion of **f**oot height to body **h**eight, 0.044 (4.4%) for men and 0.043 (4.3%) for women, stemming from 899 German men and 859 German women [25].

FH: Average diameter of the **F**emoral **H**ead, 50.94 mm for men and 44.45 mm for women, originating from radiographs of 75 male and 75 female British patients [2].

ET: Average distance from the tuberculum mediale of the **E**minentia intercondylaris to the **T**ibia plateau, 10 mm, originating from 88 specimens of human knee joints [38].

CF: Average **C**artilage thickness **F**emur within the tibiofemoral joint, 1.99 mm.

CTP: Average **C**artilage thickness **T**ibia **P**lateau, 2.92 mm.

CTPM: Average **C**artilage thickness **T**ibia **P**lateau **M**edial condyle area, 2.42 mm.

CF, CTP and CTPM stem from 3 femur specimens (1 man, 1 woman) and 12 tibia specimens (6 men, 6 women) [4].

CDT: Average **C**artilage thickness of the **D**istal **T**ibia within the talotibial joint (averaged over all measurement positions), 1.1 mm, stemming from 14 paired ankle specimens (3 male, 4 female) [5].

plt: Average **p**roportion of total (bony) **l**eg-length (TL) to **t**ibia length, 2.31 for men and 2.33 for women [24].

ptd: Average **p**roportion of **t**ibia length to **d**iameter of the tibia plateau, 4.5 for men and 4.6 for women [24].

pfe: Average **p**roportion of total **f**emur length to **e**picondyle distance, 5.9 for men and 5.8 for women [24].

TCK: Average latero-medial distance from the **T**ibia-plateau **C**entre to the **K**nee centre, 1.2 mm for men and 1.0 mm for women [24].

plt, ptd, pfe and TCK originate from full-leg-radiographs of 100 German patients, 26 male and 74 female [24].

NMC: Average distance from the ceiling of the intercondylar **N**otch to the distal end of the **M**edial femur **C**ondyle in direction of the mechanical femur axis, 9.1 mm for men and 8.2 mm for women, originating from MRIs of 50 male and 50 female healthy Chinese adults [69].

## Abbreviations

BW: Body Weight
CoM: Body Centre of Mass
EFL: External Frontal-plane Lever arm
GRF: Ground-Reaction-Force
Ht: Body Height
IFL: Knee-Internal Frontal-Plane Lever arm
IFL_LW_: Average Knee-Internal Frontal-Plane Lever arm over the stance phase of Level-Walking
IFL_SLS_: Average Knee-Internal Frontal-Plane Lever arm at static Single-Leg-Stand
IFL_TLS_: Average Knee-Internal Frontal-Plane Lever arm at static balanced Two-Leg-Stand
KAM: External Knee Adduction Moment
KAM_S_: External Knee Adduction Moment at static single-leg-stand
MA: Mechanical axis Angle. Frontal-plane angle between the line from the femoral head centre to the knee centre and the line from the knee centre to the talotibial joint centre
MFR: Medial compartment Force Ratio
