# Correction to: Towards planning of osteotomy around the knee with quantitative inclusion of the adduction moment: a biomechanical approach

**DOI:** 10.1186/s40634-021-00367-6

**Published:** 2021-06-24

**Authors:** Margit Biehl, Philipp Damm, Adam Trepczynski, Stefan Preiss, Gian Max Salzmann

**Affiliations:** 1 Fraunhofer Institute for Biomedical Engineering IBMT, Joseph-von-Fraunhofer-Weg 1, 66280 Sulzbach, Germany; 2 Charité–Universitätsmedizin Berlin, corporate member of Freie Universität Berlin, Humboldt-Universität Zu Berlin and Berlin Institute of Health, Berlin, Germany; 3 Lower Extremity Orthopaedics, Musculoskeletal Center, Schulthess Clinic, Zurich, Switzerland; 4 Gelenkzentrum Rhein-Main, Wiesbaden, Germany

**Correction to: J Exp Ortop 8:39, (2021).**

**https://doi.org/10.1186/s40634-021-00324-3**

In the original publication of this article [1], Additional files 1, 2 and 3 were incorrect. The formula in Additional file 1 must include the number “0.349” instead of “0.342”. Incorrect files have been replaced. The original article has been corrected.
