# Supplementary material for: Towards planning of osteotomy around the knee with quantitative inclusion of the adduction moment: a biomechanical approach
Source: J Exp Orthop. 2021 Jun 11;8:39. doi: 10.1186/s40634-021-00324-3 (PMC8193456; doi:10.1186/s40634-021-00324-3)
Supplement: Supplementary file 2 — Additional file 2. Influence of the tibio-femoral alignment on compartmental contact-areas and -pressures. Figure supplementing Appendix A.2. [file 40634_2021_324_MOESM2_ESM.pdf]

Towards planning of osteotomy around the knee with quantitative inclusion of the adduction moment: a biomechanical approach

Journal of Experimental Orthopaedics - DOI : 10.1186/s40634-021-00324-3

Margit Biehl, Philipp Damm, Adam Trepczynski, Stefan Preiss, Gian Max Salzmann
Margit Biehl, Diploma Physicist (Corresponding Author):
Fraunhofer IBMT, Fraunhofer Institute for Biomedical Engineering, Sulzbach 66280, Germany
e-mail: margit.biehl@ibmt.fraunhofer.de

Additional File 2:

Influence of the Tibio-Femoral Alignment on Compartmental Contact-Areas and -Pressures

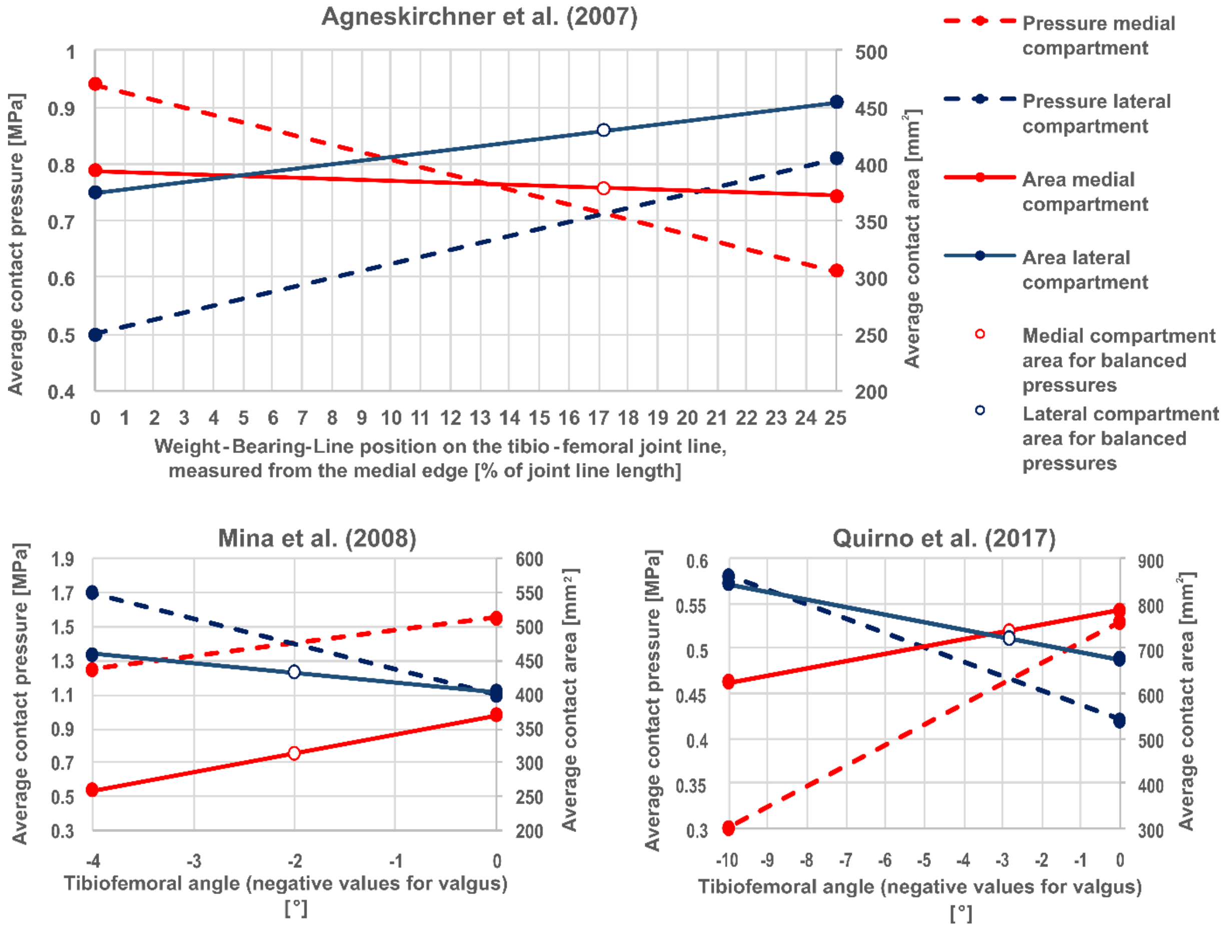

Illustration of the linear interpolation procedure to identify the axial knee force distribution for equal compartmental pressure distribution with regard to the findings of Agneskirchner et al. (2007) [1], Mina et al. (2008) [2] and Quirno et al. (2017) [3]. The solid points at the left and right borders of the graphs are corresponding to the published measurement results. The linear interpolation of average pressures in the lateral and medial compartment reveals equal compartmental pressure distribution at the intersection position of the dotted lines. The associated force in the medial and lateral compartment results from the product of the average contact pressure at this intersection position and the average medial and lateral contact area, respectively, at the same position of the horizontal axis, marked as empty circles.

References

1. Agneskirchner JD, Hurschler C, Wrann CD et al. (2007) The effects of valgus medial opening wedge high tibial osteotomy on articular cartilage pressure of the knee. A biomechanical study. Arthroscopy 23:852–861. doi: 10.1016/j.arthro.2007.05.018
2. Mina C, Garrett WE, Pietrobon R et al. (2008) High tibial osteotomy for unloading osteochondral defects in the medial compartment of the knee. Am J Sports Med 36:949–955. doi: 10.1177/0363546508315471
3. Quirno M, Campbell KA, Singh B et al. (2017) Distal femoral varus osteotomy for unloading valgus knee malalignment: a biomechanical analysis. Knee Surg Sports Traumatol Arthrosc 25:863–868. doi: 10.1007/s00167-015-3602-z
