# Supplementary material for: Towards planning of osteotomy around the knee with quantitative inclusion of the adduction moment: a biomechanical approach
Source: J Exp Orthop. 2021 Jun 11;8:39. doi: 10.1186/s40634-021-00324-3 (PMC8193456; doi:10.1186/s40634-021-00324-3)
Supplement: Supplementary file 3 — Additional file 3. Derivation of the total bony leg length from body height. Figure supplementing Appendix A.6. [file 40634_2021_324_MOESM3_ESM.pdf]

Towards planning of osteotomy around the knee with quantitative inclusion of the adduction moment: a biomechanical approach

Journal of Experimental Orthopaedics - DOI : 10.1186/s40634-021-00324-3

Margit Biehl, Philipp Damm, Adam Trepczynski, Stefan Preiss, Gian Max Salzmann
Margit Biehl, Diploma Physicist (Corresponding Author):
Fraunhofer IBMT, Fraunhofer Institute for Biomedical Engineering, Sulzbach 66280, Germany
e-mail: margit.biehl@ibmt.fraunhofer.de

Additional File 3:

Derivation of the Total bony Leg length (TL) from Body Height (Ht)

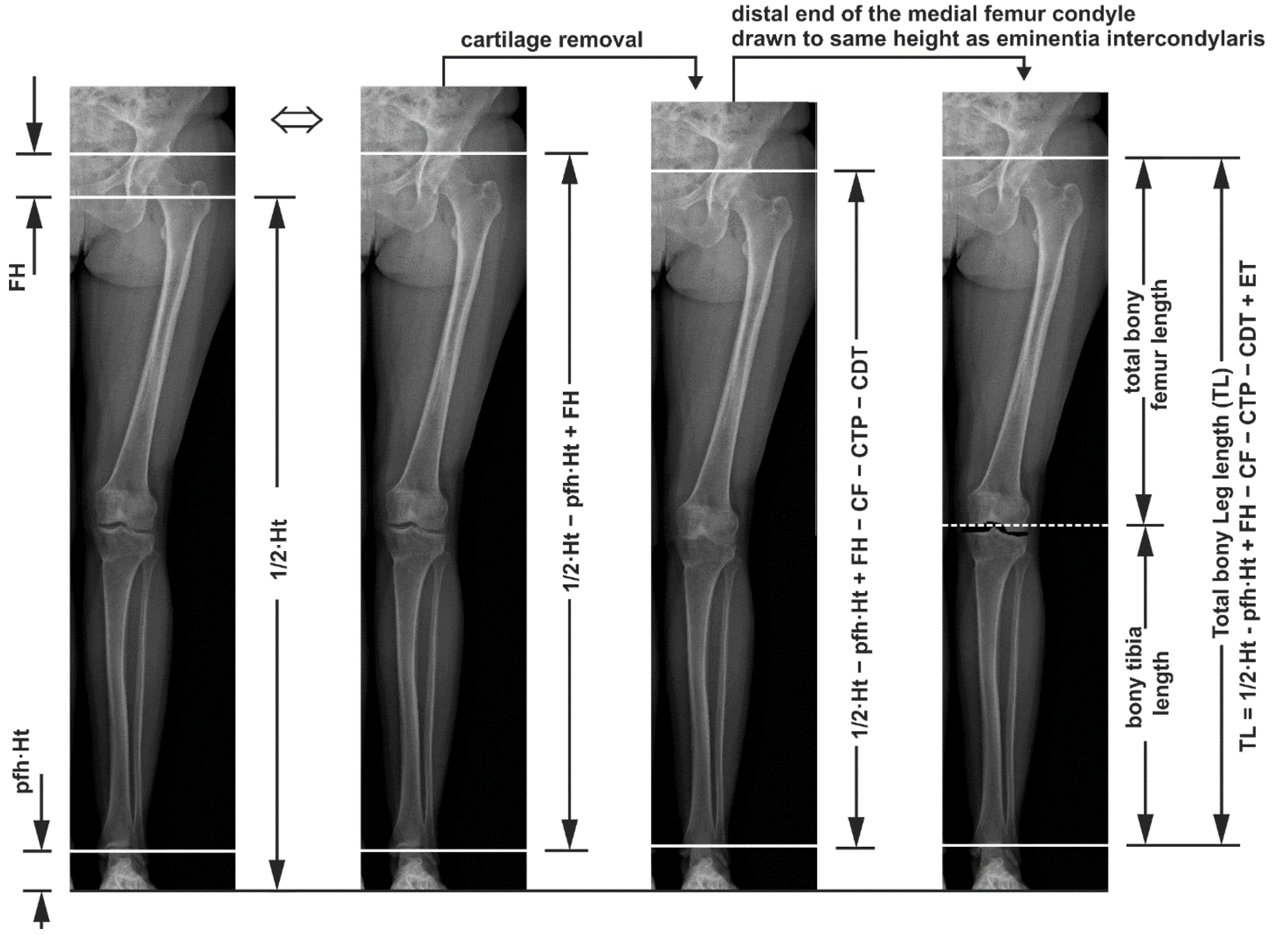

Ht: Body **H**eigh**t**. **pfh**: Average **p**roportion of **f**oot height to body **h**eight. **FH**: Average diameter of the **F**emoral **H**ead. **CF**, **CTP**, **CDT**: Average **C**artilage thickness at the distal **F**emur, **T**ibia **P**lateau and **D**istal **T**ibia, respectively. **ET**: Average distance from the tuberculum mediale of the **E**minentia intercondylaris to the medial **T**ibia plateau.
